# Utilization of Fish Skin Gelatin for Nutritional Value Enhancement of Avocado‐Based Low‐Fat Ice Cream

**DOI:** 10.1002/fsn3.4566

**Published:** 2024-11-12

**Authors:** Tanje Mada Hatsa, Dambe Genesho Jillo, Babuskin Srinivasan

**Affiliations:** ^1^ Department of Chemistry (Food and Sugar Technology Stream) Arba Minch University Arba Minch Ethiopia

**Keywords:** fish skin gelatin, food ingredient, ice cream, nutritional value, protein

## Abstract

Gelatin is one of the most widely used food ingredients, with wide applications in the food industry as stabilizing, gelling, and foaming agents. Fish skin is the basic source of gelatin, which contains a high amount of protein. The results show that the proximate compositions (protein, fat, ash, moisture, fiber, carbohydrate, and total energy) of the optimized ice cream product with ingredient compositions of (30% milk, 40% avocado pulp, 10% sugar, 15% gelatin, and 5% cream) show values of 3.26 ± 0.35, 9.32 ± 0.22, 2.79 ± 0.02, 57.83 ± 0.14, 3.46 ± 0.24, 23.26 ± 0.71, and 190.54 ± 0.02, respectively. Also, the microbe load in the optimized ice cream product was not detected up to 1 week, while total plate count and *Staphylococcus aureus* bacteria were starting to grow up after a week, and the results of panelists from sensory values indicate high acceptability of products with the aim of assessing the influence of fish skin gelatin on the nutritional values of avocado‐based low‐fat ice cream. Considering the results, gelatin has a significant effect on the nutritional and rheological properties of ice cream, specifically striking visibility on protein composition.

## Graphical abstract

Gelatin is one of the most commonly used food ingredients, with diverse uses in the food industry. In this study, fish skin gelatin is used as a nutritional enhancer in low‐fat ice cream, especially for protein reinforcement, due to the protein nature of fish gelatin. Avocado pulp is also used as a substitute for saturated fatty acids in vegetable fats.

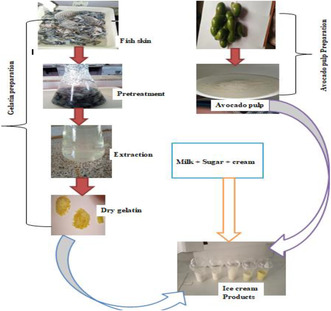

## 1 Introduction

Gelatin is made from bone and cow or pork skin; because of that, imported gelatin is still questionable of halal status (Jakhar, Anirudh, and Vardia 2016). It was reported that 41% of the gelatin produced in the world is sourced from pig skin, 28.5% from bovine skins, and 29.5% from bovine bones (Jakhar, Kumar, and Vardia 2016). It can also be made from fish skin or bones. Fish gelatin is not at risk of containing bovine sponge from encephalopathy and meets halal criteria so that it is safe for consumption by Muslims and by all people. Fish gelatin not only serves as a promising alternative to mammalian gelatin, it can also create economic value to the fish by‐products and reduce the waste generated from seafood industry. The reason for the underusage of fish gelatin is due to the presence of fishy off‐notes (Nitsuwat et al. 2021). Another important reason is the perceived inferior performance in the gelling ability of fish gelatin as compared to that of mammalian gelatin. This may be due to intrinsic factors like the chemical composition of the fish raw material, which can vary between species and parts, and environmental factors like the temperature conditions of the fish natural habitat (Wasswa, Tang, and Gu 2007). Fish gelatin has been modified to replace mammalian gelatin and applied in the yogurt production. The result suggested that fish gelatin is a promising replacement for mammalian gelatin in low‐fat stirred yogurt (Yin et al. 2021); modified fish skin gelatin was also investigated for potential applications in soft candy with the result of improving gel strength, flow behaviors, and other rheological properties of soft chocolates (Kaynarca, Gümüş, and Kamer 2023). Also, it has the potential to be used as an alternative to egg proteins to develop sustainable, low‐cost spreadable emulsions (Kaynarca 2024). The whipping properties of proteins in frozen desserts contribute to the formation of the initial air bubbles within the mix (Qiu et al. 2018). The water‐holding capability of proteins leads to an increase in viscosity in the mix, which imparts a beneficial body to the ice cream, raises the meltdown time of ice cream, and contributes to reduced iciness and sweeteners, which improve the texture and palatability of the ice cream and enhance its tastes (Goff and Hartel 2013).

Ice cream is the most essential dairy product throughout the world, and its production and consumption are rapidly increasing, and the large part of milk produced in many countries is being applied for the manufacture of frozen desserts (Me‐elahi et al. 2002). According to Choo, Leong, and Henna (2010), ice cream is a frozen and aerated dairy‐based food that is usually associated with happiness, pleasure, and fun, and psychologically, the consumption of ice cream evokes an enjoyable state for a person. It is one of the widely consumed dairy products, due to its nutritional value, and a favorite food item for a large segment of the population (Moahmmady, Abdel, and Aliaa 2019). Different functional ingredients can be added to foods to improve their quality and rheological properties, deliver health benefits, or give preferred appearances of the product structure (Zuidam and Velikov 2018). Ice cream is generally composed of a different mixture of food ingredients like milk products, sweetening materials, stabilizers, colors, flavors, emulsifiers, and eggs prepared through the agitation process (Deosarkar et al. 2016). The quality of ice cream is essentially determined by the sort of emulsifier used. The use of a proper emulsifier will produce ice cream of excellent quality (Triana et al. 2014).

In developing countries, the quality of milk and milk products is poor due to a lack of cold chain facilities, adulteration, and other malpractices involved in the handling of milk, which has a great impact on the quality of products derived from poor‐quality raw materials (Shiota et al. 2002). Several factors such as lactose intolerance, gastrointestinal discomfort, and allergies have become the principal reasons for people avoiding dairy‐based products (Ringquist et al. 2016). From a health perspective, dairy and milk‐based products such as cream and butter cause cholesterol levels (20 mg/100 g). A series of different types of non‐dairy‐based ice cream have been developed due to the rise of concerns associated with milk‐fat consumption that is dominated by clean label issues (Baars et al. 2004). Consumers prefer plant‐based diets, which are more sustainable and able to provide better health benefits (Salonen et al. 2003). Fruits and vegetables have been reported as the largest category that can promote plant‐based diets, and preference toward plant‐based diets has significantly increased with the rise of the millennial generation who are increasingly adopting vegan and free‐lactose diets (Prado et al. 2008). Particularly, avocado has a high unsaturated fat content, which is a good source for substituting the milk fat in frozen dairy desserts, and its fruits are high in phytosterol (57 mg/100 g) and total fat (15.4 g/100 g) (9.8 g/100 g) and contain zero cholesterol (Dreher and Davenport 2013).

Consequently, nowadays many fat replacers are utilized in the low‐fat frozen dessert, which will mitigate textural and sensory defects caused by reducing fat content. Fat replacers are categorized into three groups supported by their compositions: lipid‐, protein‐, and carbohydrate‐based (Akbari et al. 2019). In this regard, this study is focused on partial or complete substitutions of avocado cream in ice cream, which can be able to reduce standard fatty acid levels in ice cream and may provide significant health benefits to the customers. Not only this, but also the current study focuses on fish skin gelatin as a stabilizer and protein‐based fat replacers incorporated with plant‐based unsaturated fat content for ice cream production.

## 2 Materials and Methods

### 2.1 Sample Collection

**FIGURE 1:**
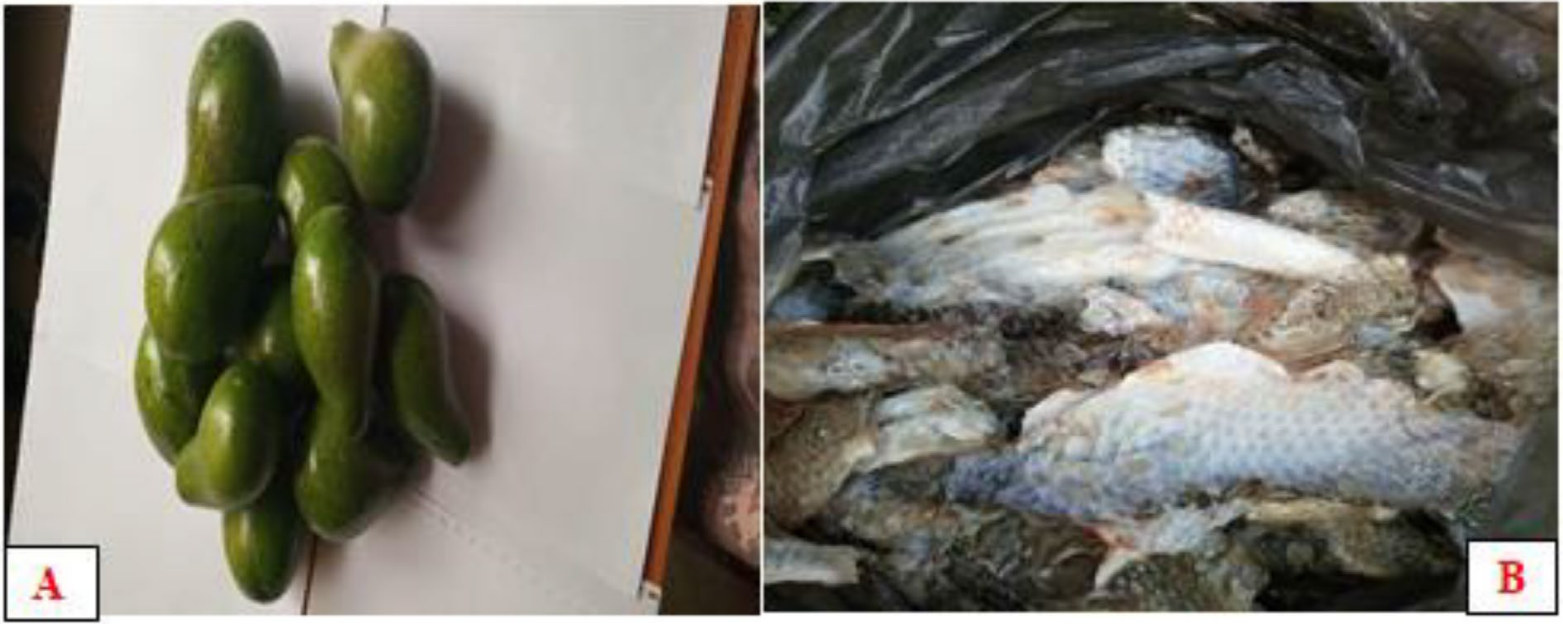
Avocado fruit sample (A), Fish skin (Nile tilapia fish skin) collected from a local fish processing factory of Lake Chamo (B).

Avocado fruit (*Persea americana*) was obtained from Lante, Arba Minch, and stored at room temperature (25°C) until the desired ripening was achieved in Figure 1A. The fish skin (*Nile tilapia* fish skin) was collected from a local fish processing factory of Lake Chamo in Figure 1B, Arba Minch, and stored in the refrigerator until use. Sugar, cream, and milk Solids‐Not‐Fat were obtained from the local market.

### 2.2 Samples Preparation

#### 2.2.1 Preparation of Gelatin From Fish Skin

**FIGURE 2:**
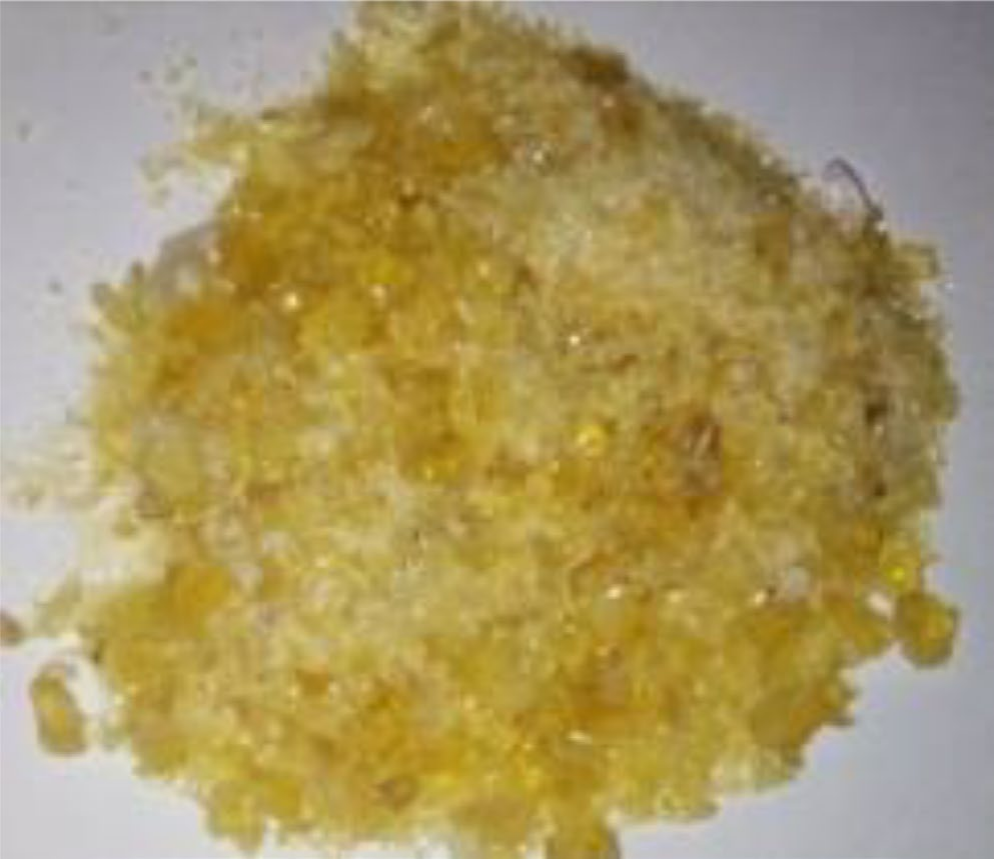
Purified gelatin extracted powder from Tilapia rendalli fish skin.

The collected *Nile tilapia* fish skins were cleaned by a scraping knife, washed, and cut into small sizes. Then, it was washed and treated with water and alkali (RANKEM, India) to remove extra material and fat and non‐collagenous protein from the scale, respectively. Then, it was soaked in 0.3% sulfuric acid (LOBA CHIME Pvt. Ltd., India) for 1 h. Also, skin was washed with tap water and had a pH of about 7.0. The skin was finally washed with distilled water to remove any residual matter. The final extraction was carried out in distilled water at 60°C and 24 h. The clear extract obtained was filtered, kept in a tray, and dried in the oven at 60°C for 16 h. The thin film of the dried material was powdered (Figure 2), weighed, packed, and stored at ambient temperature for further studies (Jakhar, Kumar, and Vardia 2016).

#### 2.2.2 Crude Protein of Fish Skin

Crude protein content was determined by method (AOAC 2005). A measure of 0.2 g of gelatin was put into Kjeldahl flask. Then 0.002 g of K2SO4 (H‐ 4 MIDC Sisco Research Laboratory Pvt. Ltd., Taloja), 0.05 g HgO (Chung Chun plastics Co. Ltd., China), and 2.5 mL of H_2_SO_4_ (C.A.S ‐5949, India) was added into the flask. The contents of the flask are then transferred into the distillation apparatus, 10 mL of concentrated NaOH was added to the apparatus until the color turned to blackish brown, and then it was distilled. The distillation product was kept in a 125‐mL Erlenmeyer containing 5 mL of H3BO3 (Chung Chun plastics Co. Ltd., China) and titrates with 0.02 N HCl (RANKEM, India), until a pink change occurred.

#### 2.2.3 Total Solid and Total Soluble Solid

The total solid and total soluble solid of the extracted gelatin was detained by the method described, with little modification (Curi et al. 2016).

#### 2.2.4 pH Determination of Gelatin

The pH of the gelatin solution was measured directly using a digital pH meter (JEMWAY 3310, United Kingdom).

#### 2.2.5 Solubility Test of Prepared Gelatin

Solubility of gelatin in the different solvents was determined by adding 0.25 g of the gelatin extract in conical flasks and shaking with 20 mL of different solvents (ethanol (Albone, Arkema, Germany), water, N‐hexane (LOBA CHEMIE Pvt. Ltd., India), and petroleum ether (Albone, Arkema, Germany)), and the solubility was determined (Mariod and Fadul 2013).

#### 2.2.6 Fourier Transforms Infrared Spectroscopy Analysis

The FT‐IR spectra of the gelatin sample were analyzed using FT‐IR spectroscopy (Perkin Elmer, spectrum 65) (Kumar et al. 2017).

### 2.3 Preparation of Avocado Pulp

Ripened avocado fruit was washed and blanched in the hot water at 75°C for 5 min to remove enzymatic brown, then dried, cut, and the avocado paste was obtained by separating the flesh of the ripe avocado from its seed and mixed using a mixer blender.

### 2.4 Physicochemical Characterization of Avocado Pulp

#### 2.4.1 Moisture and Ash Content

The moisture and ash content of avocado pulp was determined according to AOAC (2005).

$$\text{Moisture}\;\left(\%\right)=\frac{\;W1\times W2}{W1}\times 100$$

*W*_1_ = weight (g) of sample before drying, *W*_1_ = weight (g) of sample after drying.

$$\mathrm{Ash}\%=\frac{\;W1\;}{W0}\times 100$$

where W1 = weight of ash, W0 = weight of sample.

#### 2.4.2 pH Determination of Avocado Pulp

The pH of avocado pulp was measured directly using a digital pH meter (JEMWAY 3310, United Kingdom).

#### 2.4.3 Total Solids and Fiber Content in Avocado Pulp

The total solid in the avocado pulp was determined according to AOAC (2005).

$$\begin{array}{c} \text{Total solid}\;\left(\%\right)=\text{Weight}\;\mathrm{dry}\;\mathrm{pan}\\ +\text{sample}-\frac{\text{Weight}\;\mathrm{dry}\;\mathrm{pan}\;}{\text{Weight of sample}}\times 100 \end{array}$$

$$\text{Percentage of crude fiber}=\frac{W1-W2\;}{W1}\times 100$$

where W1 is the weight of the crucible, fiber, and ash, and W2 is the weight of the crucible and ash.

### 2.5 Ice Cream Preparation

Skim milk powder was first mixed with a constant amount of sugar (10%), cream (5, 10, 20%), and gelatin in different rations with four levels (0%, 5%, 10%, and 15% of gelatin) to generate a dry mix based on preliminary studies. All dry ingredients—sugar, gelatin, and milk powder—were weighed and mixed with wet ingredients with sample rations (T1 = 50% milk, 20% avocado pulp, 10% sugar, 0% gelatin, 20% cream, T2 = 50% milk, 15%avocado pulp, 10% Sugar, 5% gelatin, 20% cream, T3 = 30% milk, 40% avocado pulp, 10% sugar, 15% gelatin, 5% cream, and T4 = 40% milk, 30% avocado pulp, 10% sugar, 10% gelatin, and 10% cream) (Surjawan and Abdillah 2018). The mixtures were heated at 80°C for 5 min, followed by cooling to 4°C, and placed in ice water. Avocado fruit pulp was used in different mixing rations with four levels (15%, 20%, 30%, and 40%), and the resultant ice cream was aged for 2 h, mixed well, and frozen.

### 2.6 Proximate Composition and Physicochemical Characterization of Ice Cream Product

#### 2.6.1 Total Solids of Ice Cream Product

Total solid was determined by the gravimetric method described by Umelo et al. (2014).

$$\%\mathrm{MC}=\frac{\;W3-W2}{W2-W1\;}\times 100$$

where MC = Moisture content, W1 = Weight of empty moisture can W2 = Weight of moisture can + sample before drying.

#### 2.6.2 Crude Fat Content of Ice Cream Product

Crude fat of ice cream was measured according to AOAC (2005), and calculated as:

$$\mathrm{Fat}\;\text{percentage}=\frac{\;\text{Weight}\;\mathrm{fat}\;\text{extracted}\;}{\text{Weight of sample}}\times 100$$

#### 2.6.3 Solid Non‐Fat of Ice Cream Product

Solid nonfat was estimated by the method described by Umelo et al. (2014). It was calculated as shown below:

%Solid non−fat=%Ts−%fat.

#### 2.6.4 Crude Protein of Ice Cream Product

Crude protein content was determined following the AOAC (2005) method. A total of 0.2 g of gelatin was put into a Kjeldahl flask (CLE‐102, Bangladesh). Then, 0.002 g of K_2_SO_4_ (H‐4 MIDC Sisco Research Laboratory Pvt. Ltd., Taloja), 0.05 g HgO (Chung Chun Plastics Co. Ltd., China), and 2.5 mL of H_2_SO_4_ were added into the flask. The contents of the flask are then transferred into the distillation apparatus; 10 mL of concentrated NaOH (RANKEM, India) was added to the apparatus until the color turned to blackish brown, and then it was distilled. The distillation product was kept in a 125‐mL Erlenmeyer containing 5 mL of H_3_BO_3_ (Chung Chun Plastics Co. Ltd., China) and titrates with 0.02 N HCl (RANKEM, India), until a pink change occurred.

#### 2.6.5 Total Energy of Ice Cream Product

The total energy in the sample (kcal.) was calculated using the following equation according to AOAC (2005):

$$\text{Total energy}\;\left(\text{kcal}\right)=\left(9\times \mathrm{fat}\right)+\left(4\times \text{carbohydrate}\right)+\left(4\times \text{protein}\right)$$

#### 2.6.6 Titratable Acidity of Ice Cream Product

Titratable acidity was determined by taking 10 g of sample and placing it in a polyethylene bag and homogenized by vortex with 90 mL of distilled water added. The supernatant was filtered and the filtrate. An aliquot of 10 mL of the extract was titrated with 0.1 N NaOH (RANKEM, India) and drops of phenophtaline indicator, and color changed to a light pink at the end point (Singo and Beswa 2019).

$$\begin{array}{c} \text{Titratable acidity}\;\left(\%\text{malic acid}\right)=(\text{Volume of titer}\times 0.1\;\text{NaOH}\times \\ 0.067/\text{Volume of sample})\times 1000. \end{array}$$

#### 2.6.7 Ash and Moisture Content

Ash and moisture content were determined according to the method described by AOAC (2005).

#### 2.6.8 Crude Fiber of Ice Cream Product

The crude fiber of the ice cream samples was determined according to the methods described by Jin, Ha, and Choi (2015).

#### 2.6.9 Carbohydrate of Ice Cream Product

The total carbohydrate content was calculated by the difference among the constituents rather than analyzed directly using the following formula (Hailu 2018):

$$\begin{array}{c} \%\text{Total carbohydrate}=100-(\text{weight in grams}\;[\text{Protein}+\mathrm{Fat}\\ +\text{Moisture}+\mathrm{Ash}+\text{Fiber}]\;\text{in}\;100\;g\;\text{of sample}\;\left(\mathrm{ice}\;\text{cream}\right)). \end{array}$$

### 2.7 Determination of Microbial Dynamics

Microbial load count was measured in the colony‐forming unit based on the method described by Mada et al. (2022), with a little modification. The results of the microbial count were expressed as cfu/g of the sample by applying the following equation:

$$\mathrm{cfu}/g=\frac{\text{Number of colonies}\;\mathrm{on}\;\text{the plate}}{\text{Amount of plated sample}}\times \mathrm{Df},$$

where Df is the dilution factor.

### 2.8 Sensory Evaluation

Sensory evaluation was conducted using 50 untrained panelists distributing questionnaires and evaluating the sensory characteristics of the samples based on given parameters. Color, mouthfeel, texture, odor, and overall acceptability of samples were evaluated using a 9‐point hedonic scale, where 9—like extremely, 8—like very much, 7—like moderately, 6—like slightly, 5—neither like nor dislike, 4—dislike slightly, 3—dislike moderately, 2—dislike very much, 1—dislike extremely (Maneerat, Tangsuphoom, and Nitithamyong 2017).

### 2.9 Statistical Analysis

Statistical analysis of the data was subjected to analysis of variance (ANOVA) by using statistical software design expert 11 and Minitab version 19. The *p‐values* were used to identify significant factors, and the means are compared using the Tukey method. Significant differences between mean values were defined at the 95% confidence limit (*p* < 0.05).

## 3 Results and Discussion

### 3.1 Physicochemical Characterization of Extracted Gelatin

The variations in ash, moisture, pH, total soluble solid, total solid, and total sugar contents in extracted gelatin are described in Table 1.

**TABLE 1:** Proximate composition and physicochemical characterization of extracted gelatin.

Parameter	Values	Standard value	Reference
Moisture (%)	11.70 ± 0.82	< 15	(Europe 2000) GME requirements
Protein (%)	88.30 ± 0.81	—	—
Fat (%)	0.22 ± 0.01	—	—
TSS (%)	30.20 ± 0.17	—	—
TS (%)	88.46 ± 0.81	—	—
Ash (%)	0.37 ± 0.01	< 2	(Europe 2000) GME requirements
pH	5.9 ± 0.20	3.8–7.6	(Europe 2000) GME requirements

Abbreviations: TS, total solids; TSS, total soluble solid.

#### 3.1.1 Moisture and Ash

The moisture content of the extracted gelatin is 11.7 ± 0.82, as shown in Table 1. The result shows that the extracted gelatin was within the prescribed limit for commercial gelatin between 9% and 14%, as reported by Da Trindade et al. (2015). The present study result was similar to that of Yusof et al.'s reported result (2019) of 11.12% and 9.96% reported by Jakhar, Kumar, and Vardia. Low moisture content in gelatin increases the shelf life and affects the rheological properties of the jam incorporated into the products (Songchotikunpan, Tattiyakul, and Supaphol 2008). According to Utomo and Suryanti (2018), the moisture content in edible gelatin should be less than 15%. The result of 0.373 ± 0.015 for ash content is shown in Table 1. It was lower than that reported by Ninan, Joseph, and Aliyamveettil's (2014) recommended maximum value of 2% for edible gelatin. The relatively low ash content indicated that the extracted gelatin was of good quality (Jakhar, Kumar, and Vardia 2016). The result of ash content in the present study was reliable with the result of Nile fish skin gelatin (0.304–0.69) reported by Utomo and Suryanti (2018).

#### 3.1.2 Crude Protein

Crude protein obtained from fish skin gelatin is 88.3% ± 0.818%. The protein result in the present study was similar with the results of 83.69%, 87.24%, 88.18%, and 87.66% obtained in *Nile tilapia*, *Nile perch*, bovine reported by Badway et al. (2019), TP giant catfish (GC), and tilapia by Rawdkuen, Thitipramote, and Benjakul (2013) and Jakhar, Kumar, and Vardia (2016), respectively. Gelatin is mainly made up of protein and water (Azzainurfina, Shariffah, and Jamalulail 2019). The extracted gelatin has a high protein content, as protein is the main constituent of gelatin (Das et al. 2017).

#### 3.1.3 pH

In the present study, pH of extracted gelatin is 5.9 ± 0.2. The results obtained from fish skin gelatin are provided in Table 1. The pH of the extracted gelatin in the present study was in the pH range of the commercial gelatin (5–6), as reported by Ratnasari et al. (2014). The pH value of gelatin is influenced by the type and strength of chemicals used during the pre‐treatments (Songchotikunpan, Tattiyakul, and Supaphol 2008). The observed value was in good agreement with the study by Ojokoh (2018). The pH of fish skin gelatin was less than neutral due to acidic treatment followed while soaking to remove residual salt.

#### 3.1.4 Total Solid and Total Soluble Solid

As a result, a high total solids content (88.46% ± 0.818%) was observed in the gelatin as the gelatin samples passed through the oven for drying and turned into powder. The overall solid result of the present finding is consistent with that of a result reported by Yahdiana, Irwandi, and Effionora (2018). The total soluble solid content of the extracted gelatin is 30.2% ± 0.173%, as shown in Table 1. The sugar content and organic compounds found in the foods are represented by the total soluble solids content (Curi et al. 2016). A high value of total soluble solids in gelatin indicates a high amount of organic compounds (Yusof et al. 2019).

### 3.2 Fourier Transforms Infrared (FTIR) Characterization of Gelatin

**FIGURE 3:**
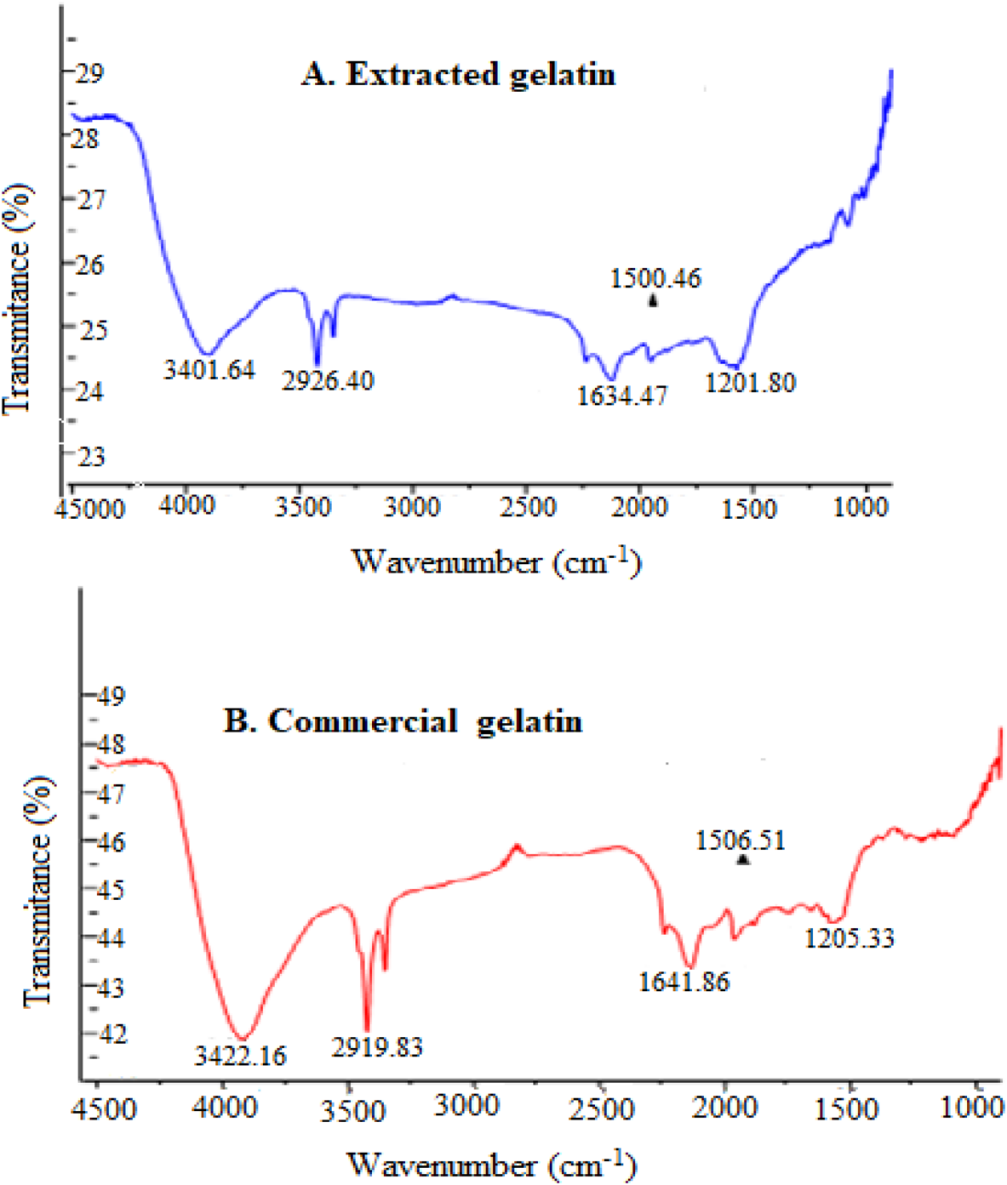
Fourier transform infrared graph of Extracted gelatin (A), and Commercial gelatin from market (B).

The FTIR analysis was used to determine the functional groups present in the fish skin gelatin molecules (Jamili et al. 2019). The functional groups in the sample are represented by the peaks, while each peak at a different wavenumber ranges between 4000 and 400 cm^−1^, and the absorption was based on the vibration mode of atoms and is very specific. The FTIR spectra of the extracted gelatin showed Figure 3A the vibration peak at the wavenumbers of 1634.47 cm^−1^ to the amide I, 1500.46 cm^−1^ to the amide II, 1201.80 cm^−1^ to the amide III, 2926.40 cm^−1^ to the amide B, and 3401.64 cm^−1^ to the amide.

The amide III of gelatin is reported to be at (1300–1000 cm^−1^) reflecting the NH bending and stretching coupled CN stretching (Cai et al. 2016). The FTIR spectra of commercial gelatin are shown in Figure 3B. The FTIR spectra of the commercial gelatin showed the vibration peak at the wavenumbers of 1641.86 cm^−1^ to the amide I, 1506.51 cm^−1^ to the amide II, 1205.33 cm^−1^ to the amide III, 2919.83 cm^−1^ to the amide B, and 3422.16 cm^−1^ to the amide A. The spectra of both commercial and extracted gelatins showed the presence of amide A at 3422.16 and 3401.64 cm^−1^, respectively, which arise from the stretching vibrations of the N–H group. Amide A band (3400–3440 cm^−1^) is related to N–H stretching vibrations (Woo et al. 2008). The amide A band reflects a free N–H stretching vibration that commonly occurs in the range of 3400–3440 cm^−1^.

### 3.3 Solubility Test of Extracted Gelatin

Gelatin swells on contact with cold water and forms large, visible swollen particles. When heated above the melting point, the hydrated gelatin bursts, goes into solution, and forms a gel when cooled (Mariod and Fadul 2013). Gelatin is almost insoluble in alcohol and the nonpolar solvents listed in Table 2. The gelatin in aqueous food systems easily forms hydrogen bonds with water due to the presence of an exposed polar region (Jamilah and Harvinder 2002).

**TABLE 2:** Solubility test of extracted gelatin.

Solution	Solubility
Water	Soluble
Ethanol	Insoluble
N‐Hexane	Insoluble
Petroleum Ether	Insoluble

### 3.4 Nutritional and Physicochemical Characterization of Avocado Pulp

Proximate composition and physicochemical characteristics of avocado pulp before preparation of ice cream are presented in Table 3.

**TABLE 3:** Proximate composition and physicochemical characterization of avocado pulp.

pH	TSS (%)	Moisture (%)	Ash (%)	Fiber (%)
6.38 ± 0.04	8 ± 0.17	78.3 ± 2.52	0.46 ± 0.29	4.95 ± 0.06

Abbreviation: TSS, total soluble solid.

#### 3.4.1 pH

The pH of avocado pulp is 6.38 ± 0.045. The avocado pulp pH is near neutral. A pH of avocado decreased depending on the duration of the storage (Bereda 2016). pH behavior is associated with the organic acid content present in the fruit since, during maturation, these tend to decrease as they are consumed at different metabolic cycles (Astudillo, Ordonez, and Rodríguez 2018).

#### 3.4.2 Fiber

The fiber content (4.95% ± 0.06%) of prepared avocado pulp is higher than the result 2.87% ± 0.00% reported by Egbuonu et al. (2018) presented in Table 3. Thus, the avocado pulp could serve as a good dietary fiber source and offers health benefits (Edem et al. 2009). Fiber improves food bulk, motility through the digestive system, appetite satisfaction, the absorption and reabsorption of cholesterol and bile acids, respectively, which could lower cholesterol levels and prevent the formation of plaque (Egbuonu et al. 2018).

#### 3.4.3 Ash Content

In the present study, ash content of avocado pulp is 0.46% ± 0.29%, as provided in Table 3. The obtained result shows that prepared avocado pulp ash content is lower than the results of 1.02 ± 0.01 and 0.78 reported by Nwaokobia et al. (2018) and Mooz et al. (2012), respectively.

#### 3.4.4 Moisture

The moisture content result from this study is 78.3% ± 2.52%. The present study result was related to the result of (78.2 ± 0.2) reported by Krumreich et al. (2018). Moisture content is one of the most important indices evaluated in foods, especially fruits, and it is a good indicator of their economic value because it reflects solid contents and serves as a tool to assess its perishable nature (Ferreira da Vinha, Moreira, and Barreira 2013).

#### 3.4.5 Total Soluble Solid

The total soluble solid obtained in the present study (8% ± 0.17%) is near to the result (8.1 ± 0.1) and higher than the result (6.68 ± 1.02) as reported by Krumreich et al. (2018). Total soluble solids content is a common quality attribute of the maturity index of agricultural products, especially fruits (Ferreira da Vinha, Moreira, and Barreira 2013).

### 3.5 Proximate Composition and Physicochemical Characterization of Ice Cream Enriched With Fish Gelatin

The proximate compositions of ice cream incorporated with different rations of gelatin in ice cream preparation are presented in Table 4.

**TABLE 4:** Proximate composition of ice cream incorporated with different ratios of gelatin containing milk, avocado pulp, sugar, and cream.

Ice cream Code	Protein (%)	Fat (%)	Ash (%)	Moisture (%)	Fiber (%)	Carbohydrate (%)	Total energy (Kcal)
T1	2.94 ± 0.03^c^	11. 82 ± 0.54^a^	2.47 ± 0.075^a^	53.35 ± 1.16^a^	0.27 ± 0.0^d^	27.41 ± 1.50^a^	236.91 ± 4.01^a^
T2	3.09 ± 0.10^b^	10.07 ± 0.22^b^	2.71 ± 0.06^ab^	55.98 ± 0.1^b^	1.99 ± 0.04^c^	25.06 ± 0.65^ab^	211.20 ± 0.915^b^
T3	3.26 ± 0.13^a^	9.38 ± 0.22^c^	2.79 ± 0.02^b^	57.83 ± 0.14^c^	3.46 ± 0.2^b^	23.26 ± 0.71^bc^	190.54 ± 0.53^c^
T4	3.07 ± 0.07^b^	9.70 ± 0.06^c^	2.65 ± 0.05^c^	59.81 ± 0.28^d^	4.34 ± 0.17^a^	21.55 ± 0.43^c^	176.27 ± 1.81^d^
*p*	*p* < 0.05	*p* < 0.05	*p* < 0.05	*p* < 0.05	*p* < 0.05	*p* < 0.05	*p* < 0.05

*Note:* Values with different superscript letters (a, b, c, d, ab, bc) within the same column are significantly different (*p* < 0.05) *as per Tukey test ( Mada et al. 2022)*. T1= 50% milk, 20% avocado pulp, 10% Sugar, 0% gelatin, 20 % cream Total = 100%, T2= 50% milk, 15% avocado pulp, 10% Sugar, 5% gelatin, 20% cream Total = 100%, T3= 30% milk, 40% avocado pulp, 10% Sugar, 15% gelatin, 5 % cream, Total = 100 %.

#### 3.5.1 Ash Content

The results of ash content in ice creams are 2.437% ± 0.0745%, 2.710% ± 0.060%, 2.793% ± 0.025%, and 2.653% ± 0.0057% for T1, T2, T3, and T4 in Table 4. The results of the mean show a statistically significant difference between T1 and T2, T3, and T4 (*p* < 0.05). A significant difference was also found between T3 and T4 (*p* = 0.035). T3 has the highest ash content at 2.79 ± 0.025, followed by (T2) 2.71 ± 0.06, (T4) 2.65 ± 0.057, and (T1) 2.47 ± 0.075, respectively. In T3, the increase in ash was due to the presence of a large amount of dry gelatin stabilizer powder. The ash content result in the present study was high compared to the result of Lima, Brito‐oliveira, and Pinho (2016). The differences in ash content were mainly due to the different proportion of the mineral in the raw material used in the formulation.

#### 3.5.2 Moisture Content

The present experiment moisture values were analyzed statistically and show that the mean value between T1 and T3 (*p* = 0.05) and T4 (*p* = 0.001) is a significant difference. In the same way, a significance difference was also found between T2 and T4 (*p* = 0.027). The values between T1 and T2 (*p* = 0.150), T2 and T3 (0.122), and T3 and T4 (*p* = 0.707) were not statistically significant. This implied that the avocado contributed more to the moisture in the ice cream samples than the milk and cream with respect to the ingredient used. The moisture content of ice cream is generally high due to the milk content in the mixture of products. The moisture content of this finding was in the range with the result in a finding of Omayma and Youssef (2014).

#### 3.5.3 Protein Content of Ice Cream Product

The protein result values of the current studies are 2.94% ± 0.031%, 3.09% ± 0.100%, 3.26% ± 0.135%, and 3.07% ± 0.0755% for T1, T2, T3, and T4. There is a statistically medium variation between all treatments (*p* < 0.05). It can be observed that the protein content in T3 was higher when other treatments were applied. The protein increases in ice cream are directly related to the amount of stabilizer (gelatin). Due to fish skin, gelatin is the basic source of protein (Jaswir et al. 2016). However, the protein content in the product from T1 and T4 decreases as avocado inclusion increases while milk and whipped cream inclusion decreases. The control sample (T1) had the lowest protein content (2.94% ± 0.031%), followed by T4 and T2. Even if the control sample does not contain gelatin in the product formulation, it has protein content since milk itself is one of the protein sources in nature. Increasing the gelatin content increases the protein content in ice cream. This emerges from a recent study, which is supported by the results of a previous outcome study (4.03, 3.71–4.25, 3.345–3.430) reported by Chavan et al. (2017), Çam et al. (2013), and Abdullah et al. (2003).

#### 3.5.4 Fat Content of Ice Cream Product

The fat content of ice cream decreased as avocado inclusion increased. The mean results for fat content of ice creams are 11.270% ± 0.540%, 10.07% ± 0.22%, 9.38% ± 0.23%, and 9.700% ± 0.060% for T1, T2, T3, and T4, respectively. Statistical analysis shows that significant differences were found between treatments T1, T2, T3, and T4 (*p* < 0.05). The highest fat content was observed at T1 (11.82% ± 0.54%) and the lowest fat content was observed at T3 (9.38% ± 0.06%). The decrease in the fat content of ice cream was due to the reduction in the fat content of dairy cream, which was subsequently replaced by avocado. Fat content contributes to fat crystallization and could influence the textural properties of ice cream. An increase in fat content shows an increase in melting rate, with melting rate being lower for low‐fat ice cream (Syed et al. 2018). The fat contents are in line with the recommendation to consume foods with a low‐fat composition for a healthier diet.

#### 3.5.5 Fiber Content of Ice Cream Product

From present studies, the fiber contents are 0.278% ± 0.0192%, 1.996% ± 0.0416%, 3.463% ± 0.240%, and 4.340% ± 0.170% for T1, T2, T3, and T4, respectively. The highest fiber content was observed in (T4) 4.34 ± 0.170, followed by (T3) 3.46 ± 0.24, (T2) 1.99 ± 0.042, (T1) 0.278 ± 0.019, respectively. Dietary fiber in ice cream promotes an effective control of ice crystallization and ice crystal development during freezing and storage, as well as improves the melting and viscosity properties for the ice cream products (Staffolo, Bertola, and Martino 2004; Soukoulis, Lebesi, and Tzia 2009). The result of the fiber in the present study is lower than the result (5.14) reported by Surjawan and Abdillah (2018). The avocado pulp could retain the melting rate of the ice cream due to the polysaccharides and natural dietary fiber content in it. A higher concentration of avocado pulp in the ice cream formula provides more hydrocolloids.

#### 3.5.6 Carbohydrate of Ice Cream Product

The main source of carbohydrates is sugars, which provide a useful source of energy for cells (Deosarkar et al. 2016). The carbohydrate contents of the ice cream samples are 27.417% ± 1.506%, 25.06% ± 0.65%, 23.26% ± 0.71%, and 21.55% ± 0.427% for T1, T2, T3, and T4, respectively. Statistical analysis showed that significant differences were found between T1 and T3 (*p* = 0.002) and T4 (*p* < 0.050). A significant difference was also found between T2 and T4 (*p* = 0.007). The carbohydrate content of the ice cream was highest in the T1 sample with a mean of 27.42% ± 1.51%, while T4 had the lowest value with 21.55% ± 0.427%. The result value was consistent with that of reported results of Umelo et al. (2014) (20, 29–24, 17) and Pankiewicz et al. (2020) and Silva and Lannes (2011) (25.43–27.21), and higher than that of Deosarkar et al.'s (2016) reported result.

#### 3.5.7 Total Energy of Ice Cream Product

The energy value of food represents the contribution that food makes to the total energy requirements of the body. Ice cream is an important source of food energy. The fact that the ingredients of ice cream are almost completely assimilated makes ice cream an especially desirable food for growing children and for persons who need to maintain or put on weight, for example, the elderly (Arbuckle 2013). The total energy values are 236.91 ± 4.01, 211.20 ± 0.915, 190.54 ± 0.534, and 176.27 ± 1.81 for T1, T2, T3, and T4, respectively, as provided in Table 4. The statistical analysis indicates the total energy value is significantly different (*p* < 0.05) among the treatments. The result of the present study is in line with the result of (196.7–377) reported by Deosarkar et al. (2016).

### 3.6 Physicochemical Properties of Ice Cream Products

The physicochemical properties of avocado pulp‐incorporated ice cream are presented in Table 5.

**TABLE 5:** Physicochemical characteristics of ice cream products with samples.

Ice cream	pH	TA (%)
T1	6.62 ± 0.02^a^	0.03 ± 0.07^b^
T2	6.59 ± 0.01^a^	0.05 ± 0.05^ab^
T3	6.58 ± 0.03^a^	0.05 ± 0.01^ab^
T4	6.60 ± 0.06^a^	0.06 ± 0.04^a^

*Note:* Means in the same column indicated by different letters (a, b, ab) were significantly different (*p* < 0.05) ( Mada et al. 2022). T1= 50% milk, 20% avocado pulp, 10% Sugar, 0% gelatin, 20 % cream Total = 100%, T2= 50% milk, 15% avocado pulp, 10% Sugar, 5% gelatin, 20% cream Total = 100%, T3= 30% milk, 40% avocado pulp, 10% Sugar, 15% gelatin, 5 % cream, Total = 100 %, T4= 40% milk, 30%avocado pulp, 10% Sugar, 10% gelatin, 10% cream, Total = 100%.

Abbreviation: TA, titratable acidity.

#### 3.6.1 pH

The present study results of pH values are 6.62 ± 0.02, 6.59 ± 0.01, 6.58 ± 0.03, and 6.60 ± 0.06 for T1, T2, T3, and T4, respectively, comparable with the result of 6.5 reported by Khalil and Blassy (2015) and Surjawan and Abdillah (2018). The pH of all the treatments was near‐neutral pH and rage of the acceptable pH range for ice cream (Umelo et al. 2014). There were no significant differences (*p* < 0.05) observed in pH values of ice cream samples.

#### 3.6.2 Titratable Acidity of Ice Cream Product

A statistically significant difference was found between T1 and T4 (*p* < 0.05). While significant differences were found between T1 and T2 (*p* = 0.557), T3 (*p* = 0.370), T2 and T3 (*p* = 0.981), T4 (*p* = 0.220), and T3 and T4 (*p* = 0.355), respectively. It was observed that the acidity of treatment T1 (0.037% ± 0.007%) was lower when compared to other treatments, and the acidity of T4 (0.065% ± 0.014%) was higher. A higher acidity or lower pH is undesirable as it contributes to the excess viscosity, decreased whipping, inferior flavor, and less stable mix leading to its coagulation during processing (Bajad et al. 2016). The acidity values obtained for ice cream samples in this study were comparable with the results of (0.15–0.33) reported by Çam et al. (2013).

#### 3.6.3 Microbial Analysis

The total plate count of *Salmonella*, *Shigella*, *E. coli*, and *Staphylococcus aureus*; were examined to quantify the presence of microorganisms in food samples.

In the current study, the total plate count, *E. coli*, *Staphylococcus aureus*, *Salmonella*, and *Shigella* were not countable for seven days. The increment of microbial count of any food indicates poor hygienic conditions prevailing, while increase of *Staphylococcus aureus* indicates unclean conduct, which may cause numerous problems like diarrhea, dysentery, and vomiting that come from the skin, nose, hands, and clothes of the handler (Pal et al. 2012; Masud 1989). Results of the current study were at a safe level in Table 6 when compared with the results of ice cream sold in the market, which is unsatisfactory for human consumption and poses a major health hazard to the public. According to Masud's (1989) report, the situation could be resolved successfully by avoiding using unstandardized product and embarking on vigorous quality control. Most ice creams become contaminated with microbes during processing, transportation, and preservation, and contaminated food products can be responsible for food‐borne infections in children and elderly people (Jadhav and Raut 2014). The current finding result of *E. coli* was lower and *Staphylococcus aureus* was higher than the finding of Fadihl, Mohammad, and Al‐qrtani (2019). In the current study, the presence of microbial load was very low. This indicates that the ice cream preparation process, place, and personal hygiene were at a safe level and ice cream containers were packed well.

**TABLE 6:** Microbial load analysis of ice cream products after 1 week.

Treatment	TPC (cfu/g)	*Salmonella* (cfu/g)	*Shigella* (cfu/g)	*E. coli* (cfu/g)	*Staphylococcus aureus* (cfu/g)
T1	24 × 10^2^	ND	ND	7 × 10^2^	11 × 10^2^
T2	29 × 10^2^	ND	ND	11 × 10^2^	10 × 10^2^
T3	41 × 10^2^	ND	ND	6 × 10^2^	22 × 10^2^
T4	53 × 10^2^	ND	ND	ND	31 × 10^2^

Abbreviations: cfu/g, colony‐forming unit per gram; ND, not detected; TPC, total plate counts T1= 50% milk, 20% avocado pulp, 10% Sugar, 0% gelatin, 20 % cream Total = 100%, T2= 50% milk, 15% avocado pulp, 10% Sugar, 5% gelatin, 20% cream Total = 100%, T3= 30% milk, 40% avocado pulp, 10% Sugar, 15% gelatin, 5 % cream, Total = 100 %, T4= 40% milk, 30% avocado pulp, 10% Sugar, 10% gelatin, 10% cream, Total = 100%.

#### 3.6.4 Sensory Evaluation of Ice Cream Products

**FIGURE 4:**
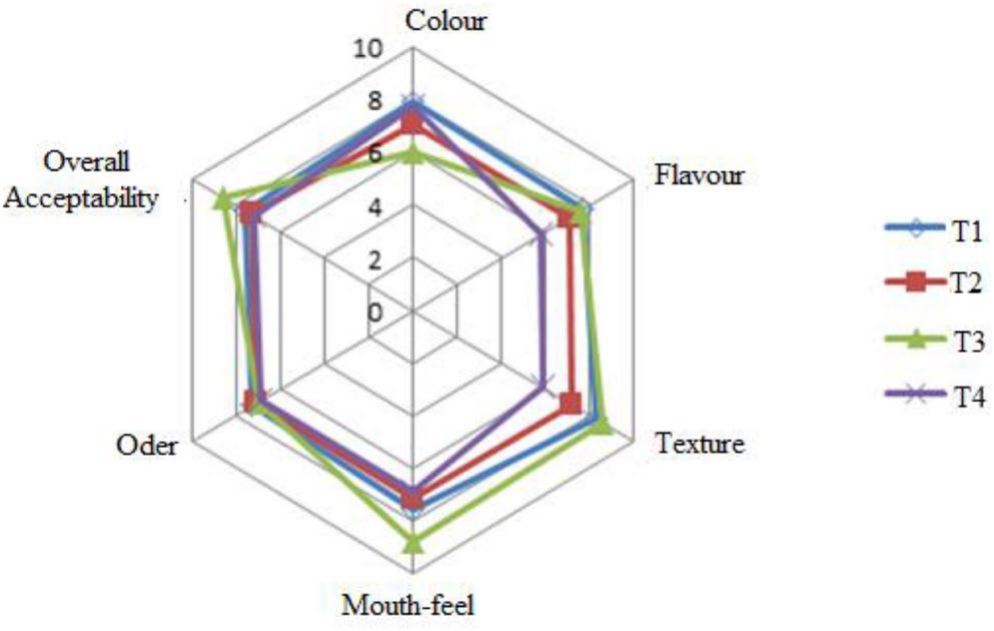
Sensory properties of ice cream samples containing milk, avocado pulp, sugar, and cream. T1 contains 50% milk, 20% avocado pulp, 10% sugar, 0% gelatin, and 20% cream; T2 contains 50% milk, 15% avocado pulp, 10% sugar, 5% gelatin, and 20% cream; T3 contains 30% milk, 40% avocado pulp, 10% sugar, 15% gelatin, and 5% cream; T4 contains 40% milk, 30% avocado pulp, 10% sugar, 10% gelatin, and 10% cream.

Sensory properties, especially color, mouthfeel, taste, texture, smell, and general acceptability of dairy products, are important quality characteristics and are considered the most critical indicators of consumer performance (Mada et al. 2022). The panelists' results from the web diagram in Figure 4 show that the sample code T3, composed of 30% milk, 40% avocado pulp, 10% sugar, 15% gelatin, and 5% cream with a value of 6.01, has a lower color value. However, most of the sensory attributes, texture, mouthfeel, and overall acceptability were higher with scores of 8.55, 8.72, and 8.55, respectively.

From this result sample, Code T3 contains a large amount of gelatin, which is considered a good stabilizing agent for rheological properties in ice cream products. Example code T1 (50% milk, 20% avocado paste, 10% sugar, 0% gelatin, and 20% cream) shows results from test participants with higher values of 7.86 in color. From this point of view, for the other samples, the color of the ice cream becomes dark green as the amount of avocado pulp and gelatin increases. The taste of all samples is based on the panelists' ratings and is almost identical.

## 4 Conclusions

In this study, avocado is incorporated with gelatin, cream, and skim milk powder to prepare low‐fat ice cream. The proximate composition and physicochemical properties of avocado fruit, fish gelatin, and prepared ice cream are evaluated. Gelatin was used as a protein enhancer and texture modifier in prepared ice cream. In avocado pulp, unsaturated fatty acids substitute the milk fat (saturated fatty acid in the milk) with cream in various combinations of milk (30, 40, 50), avocado (15, 20, 30, 40), gelatin (0, 5, 10, 15), constant amount of sugar (10%), and cream (5, 10, 20) to improve the quality of ice cream. The physicochemical properties of ice cream such as total soluble solids decreased and titratable acidity increased with increasing avocado integration. The approximate composition of low‐fat avocado‐based ice cream, such as fat, carbohydrates, and total energy content, were decreased, while protein and fiber of the ice cream increased with increasing gelatin and avocado incorporation. The sensory properties of ice cream (color, flavor, odor, mouthfeel, texture, and overall acceptability) were evaluated by 50 untrained panelists. Panelist state that the color of ice cream changed from white to green as avocado concentration increased, and milk flavor also changed into the avocado flavor. There was a significant difference in overall acceptability between samples with 15%, 20%, 30%, and 40% of avocado replaced with milk fat. Microbial growth such as total bacterial counts, *E. coli*, *Staphylococcus aureus*, *Salmonella*, and *Shigella* could not be counted for a week, while total bacterial counts and *Staphylococcus aureus* began to grow after a week. Therefore, fish skin gelatin and avocado pulp can be successfully used to make nutritionally rich and low‐fat ice cream.

## Author Contributions

**Tanje Mada Hatsa:** data curation (equal), visualization (supporting), writing – original draft (equal). **Dambe Genesho Jillo:** conceptualization (lead), methodology (lead), writing – original draft (equal). **Babuskin Srinivasan:** project administration (lead), resources (equal), software (supporting), supervision (lead), validation (supporting).

## Ethics Statement

The authors have nothing to report.

## Conflicts of Interest

The authors declare no conflicts of interest.

## Data Availability

All necessary data are provided in the article. Nevertheless, the authors agree to share raw data upon request.
